# The analysis of residential sorting trends: Measuring disparities in socio-spatial mobility

**DOI:** 10.1177/0042098018798759

**Published:** 2018-11-27

**Authors:** Tal Modai-Snir, Pnina Plaut

**Affiliations:** OTB – Research for the Built Environment, Faculty of Architecture and the Built Environment, Delft University of Technology, The Netherlands; Faculty of Architecture and Town Planning, Technion – Israel Institute of Technology, Israel

**Keywords:** residential mobility, residential sorting, segregation, socio-spatial mobility, sorting trends, spatial level

## Abstract

Ethnic and socioeconomic segregation levels vary over time and so do the spatial levels of these segregations. Although a large body of research has focused on how residential mobility patterns produce segregation, little is known about how changing mobility patterns translate into temporal and scale variations in sorting. This article develops a methodological framework designed to explore how changing mobility patterns reflect such trends. It introduces a measure of sorting that reflects the extent of disparities among groups in their socio-spatial mobility. Trends in the direction and the extent of sorting can be exposed by computing sorting measures over consecutive periods. The measure is broken down to capture the relative contributions of residential mobility to sorting at hierarchically nested geographical units, for example cities and their constituent neighbourhoods. An empirical demonstration shows that changes in residential mobility patterns affect the magnitude and spatial level of residential sorting, which vary even over the short term.

## 摘要

种族和社会经济隔离水平随着时间的推移而变化，这些隔离的空间水平也是如此。虽然大量的研究都集中在居住流动模式如何产生隔离，但对于流动模式的变化如何转化为分类的时间和规模变化知之甚少。本文开发了一个方法框架，旨在探索不断变化的流动模式如何反映这些趋势。它引入了一种分类指标，反映了群体在社会空间流动方面的差异程度。通过计算连续时期的分类指标，可以暴露出分类方向和程度的趋势。该指标被分解以捕获居住流动性对分层嵌套地理单元（例如城市及其组成街区）的分类的相对贡献。实证证明，居住流动模式的变化会影响居住分类的规模和空间水平，即使在短期内也会出现不同。

## Introduction

The levels of economic and ethnic segregation vary over time (Bischoff and Reardon, 2013; Glaeser and Vigdor, 2012) and so do the spatial levels of these segregations; evidence points to increasing segregation at the inter-city at the expense of the inter-neighbourhood level (Fischer et al., 2004; Lichter et al., 2015b; Massey et al., 2009; Reardon et al., 2009). Despite the long tradition of analysing residential mobility patterns to understand the mechanisms underlying segregation, this line of research hasn’t engaged with investigating how changing mobility patterns translate into the observed segregation trends. There is no evidence on how changing residential choices generate trends in the extent of sorting, and it is still not understood how the intersecting process of selecting cities and neighbourhoods within cities leads to scale-trends in sorting.

The common practices in residential mobility research do not account for the changing extent and scale of sorting. Often, they include an estimation of different groups’ mobility probabilities among neighbourhood types to show how they diverge. Current practices do not quantify this divergence and hence cannot compare the effect of mobility on sorting over time; they do not deal with how the changing divergence in mobility patterns is structured across hierarchical spatial levels, and so the changing effect of mobility on the scale of sorting cannot be indicated.

In this article we define a measure of sorting that quantifies the extent of divergence in observed mobility patterns of two groups with respect to a single place attribute. It can be followed over time and can be broken down to components that indicate the relative contribution of residential mobility to sorting at hierarchically-nested geographical levels, for example cities and their constituent neighbourhoods. It builds on a ‘socio-spatial mobility’ approach which regards residential moves as a social repositioning that stems from the change in neighbourhood attributes following relocation. The introduced framework can be applied in different contexts of residential segregation: ethnic, racial and socioeconomic.

## Background

Residential mobility is a readjustment process designed to maximise households’ utility by matching the characteristics of housing to the household’s needs and aspirations; it is triggered by a certain *mismatch* between a household and its current dwelling, and it is directed towards creating a *better match* (Hanushek and Quigley, 1978). This matching process involves multiple characteristics of both households and dwellings. Household factors that shape housing needs and aspirations are, for example, socioeconomic status, life-cycle stage and ethnic identity (Clark and Dieleman, 1996). Dwellings are evaluated by households according to their structural characteristics (for example, size or quality) and locational features such as accessibility, amenities and the neighbourhood makeup. The act of matching household aspirations and neighbourhood makeups is the basic manifestation of the residential sorting process, the most powerful mechanism of segregation.

Residential mobility patterns change over time and can generate trends in sorting (Crowder and South, 2005) due to diverse processes. For example, immigrants’ social mobility and adaption can translate into residential choices that reflect higher spatial integration (e.g. Lichter et al., 2015a; Massey and Denton, 1985; South et al., 2005). Public attitudes towards minorities can change over time (e.g. Semyonov et al., 2006) and affect the residential choices of majority members in relation to minority composition. Finally, mobility patterns are affected by urban areas’ structural characteristics (South et al., 2011); changes in structural characteristics (for example, population composition and inequality levels) are therefore expected to alter mobility patterns and sorting. These examples demonstrate the focus of this article on aggregate-level change in mobility patterns, which reflects societal and macroeconomic change; as a longitudinal inquiry it should be distinguished from studies that focus on within-individual residential trajectories related to the life-course (e.g. South et al., 2016).

Trends in the spatial level of sorting can be driven by additional factors related to mobility. A changing spatial typology of mobility can induce such a trend; the larger the proportion of inter-city compared with intra-city relocations, the larger the potential contribution to sorting at the inter-city level. A changing preference for relative positioning can also affect the scale of sorting; for example, increasing preference for low-positioned neighbourhoods in high-positioned cities against the opposite situation. Finally, the existing hierarchical socio-spatial structure is not only shaped by mobility patterns but also shapes them such that historical patterns tend to be reinforced. The variation among neighbourhoods within cities, compared with the variation among cities, determines the dominance of sorting at one level over the other. The stronger the spatial autocorrelation (i.e. neighbourhoods within each city relatively similar to each other, and cities, vary greatly among themselves), the more dominant the residential sorting at the higher spatial level.

To explain observed segregation patterns, a common practice in residential mobility studies is to examine how different groups diverge in probabilities of making moves among neighbourhood categories. In the ethnic context, studies examined how majority and minority members differ in relocation patterns among places characterised by their majority/minority composition (e.g. Bolt et al., 2008; Hall and Crowder, 2014; Quillian, 2002; South and Crowder, 1998). In the socioeconomic context, studies examined how groups diverge in their relocation among neighbourhoods of different socioeconomic contexts (e.g. Crowder and South, 2005; Gramlich et al., 1992; South and Crowder, 1997). These studies point to diverging patterns among groups but do not indicate the extent of sorting and its change over time. Several modelling strategies have been used to understand how observed mobility probabilities translate into segregation, including agent-based models (e.g. Clark and Fossett, 2008) and Markov models (e.g. Tuljapurkar et al., 2008). While they do link diverging mobility patterns to distributional outcomes, they only rely on patterns observed at a single time point. Some of them account for endogenous change in moving probabilities that arises from the changing characteristics of places due to mobility, but none of them account for trends related to exogenous social and economic change. Another approach characterises mobility patterns as the extent of change in neighbourhood attribute values that movers experience as a result of relocation. This has been termed ‘socio-spatial mobility’, which conveys the idea of movement across the social hierarchy of places (Clark and Morrison, 2012; Clark et al., 2014). The ‘socio-spatial mobility’ approach enables observation of moves across the full hierarchy of neighbourhoods and not only among specific neighbourhood classifications. As a result, it does not rely on arbitrary cut-offs to represent neighbourhood types,^1^ and mobility patterns form a continuum. Change in neighbourhood socioeconomic contexts has been expressed as the arithmetic difference between destination and origin indicator values (Clark et al., 2014; Modai-Snir and Plaut, 2015), also relative to origin values (Clark and Morrison, 2012), or as difference between destination and origin quantile positions (Brazil and Clark, 2017; Clark and Morrison, 2012; Clark et al., 2014).^2^ These measures correspond to measures used in social and income mobility research, which focuses on the changing social and economic positions of individuals over time. In fact, they adhere to different mobility ‘concepts’ which were defined in that context (Fields, 2008). The former corresponds to what Fields (2008) identified as the concept of ‘directional movement’ (mobility as movement across absolute positions within the distribution). The latter corresponds to the concept of ‘positional movement’.

Studies using both the ‘probability’ and ‘socio-spatial mobility’ approaches have not examined the divergence in mobility patterns over time. Both approaches to residential mobility analysis have been applied so far to a single spatial level. Residential choices have been observed as moves between neighbourhoods regardless of the fact that they reflect a multilevel choice, for example of a specific neighbourhood within a specific city. The framework developed in this article will bridge these gaps by addressing the following questions: How does the divergence in mobility patterns change over time? And how is this divergence structured across hierarchical spatial levels?

## Methodological framework

The framework presented in this article develops a measure of sorting which can be followed longitudinally to represent trends and broken down to reflect the changing spatial levels at which sorting occurs. Its development is based on a ‘socio-spatial mobility’ approach which assesses the direction and extent of changes in neighbourhood attributes that are experienced by movers as a result of relocation. It is also based on the rationale that the extent of sorting among groups can be represented by the degree of divergence in their socio-spatial mobility patterns.

Conceptually, residential sorting is the act of matching people’s attributes to those of place makeups. Sorting occurs when the nature of changes in place characteristics resulting from moving creates better matches with movers’ attributes. For example, income sorting occurs when rich people move from poor to rich neighbourhoods and poor people move from rich to poor neighbourhoods. Racial sorting occurs when black people move from ‘white’ to ‘black’ neighbourhoods and white people move from ‘black’ to ‘white’ neighbourhoods. In reality, place characteristics present wide distributions. Relocation patterns may be analysed, therefore, as moves to ‘richer’, ‘poorer’, ‘blacker’ or ‘whiter’ destinations, relative to origins. The relation between origin and destination characteristics is defined as ‘socio-spatial mobility’.

To indicate how much sorting occurs as a result of aggregate relocations, it is necessary to observe how groups of movers move in relation to each other. The fact that rich movers move to richer places is not sufficient to indicate whether the mobility structure reflects socioeconomic sorting; if poor people made similar moves at a certain period, then aggregate mobility patterns would not reflect sorting (over and above existent spatial patterns). The development of a sorting measure, then, is based on quantifying the disparities among mover-groups in their socio-spatial mobility patterns, while attributes of movers are examined in relation to a corresponding neighbourhood attribute. Socioeconomic sorting is examined by observing disparities in income groups’ socio-spatial mobility with respect to neighbourhood socioeconomic characteristics. Ethnic sorting is examined by observing disparities among majority and minority members in their mobility among neighbourhoods characterised by minority/majority composition. This conceptual framework can be applied to other spatial units, such as cities.

Spatial distributions are also affected by those who do not move. The framework presented here deals only with the sorting associated with relocations and exposes, therefore, the ‘marginal effect’ of moving behaviours on sorting.

### A measure of socio-spatial mobility

To quantify the socio-spatial mobility of groups we have chosen to use a ‘directional mobility’ measure which was proposed by Fields and Ok (1999) in the context of income mobility (equivalent to that used in the socio-spatial context by Clark and Morrison, 2012; Clark et al., 2014; Modai-Snir and Plaut, 2015) and conforms to an ‘absolute’ concept of mobility. This concept captures the extent of change associated with ‘real’ place conditions that can alter over time for similar place relative positions.^3^

We define the ‘*per-capita socio-spatial mobility*’ measure which represents the marginal mobility of a mover from group *a* with respect to a single neighbourhood attribute:

(1) $$M_{a}=\frac{1}{n}\sum_{i=1}^{n}\left(d_{i}-o_{i}\right)$$

where $M_{a}$ is the average mobility of sub-group *a*, and *d_i_* and *o_i_* refer to neighbourhood attribute values in the destination and origin of each individual relocation *i*, respectively; *n* refers to the total number of movers in this sub-group.

To illustrate, assume that socioeconomic sorting is examined by analysing the mobility of different mover income-groups with regard to neighbourhood socioeconomic status, represented by its *percentage of high-income residents*. A relocation of person *a* from a neighbourhood with 10% high-income residents to one with 15% yields a mobility of 5 *pp* (*percentage points*). A relocation of person *b* from a neighbourhood with 20% high-income residents to one with 30% yields a mobility of 10 *pp*. If we quantified mobility relative to origin status, as in Clark and Morrison (2012), these examples would reflect the same degree of mobility.

### A measure of sorting

Assume that person *a* is poor and that person *b* is rich. The degree of socioeconomic sorting reflected in both their relocations may be quantified as 5 *pp*, which is the difference between the extent of socio-spatial mobility each of them experienced. This figure reflects the extent to which rich and poor diverge in the attainment of neighbourhood socioeconomic status. While the illustration refers to a society of two persons, the procedure averages the socio-spatial mobility measures of all movers of each sub-group. We define the measure of *differential mobility* as the arithmetical difference in the socio-spatial mobility levels of two groups:

(2) $$DM_{\left(a,b\right)}=M_{a}-M_{b}$$

where $DM_{\left(a,b\right)}$ denotes a differential-mobility measure relating to mover sub-groups *a* and *b*, and *M* denotes a mobility measure as described in eq. 1. The interpretation of the resultant value depends on the order of groups conjointly with the neighbourhood variable chosen. In the case of neighbourhood compositional variables (such as the percentage of neighbourhood residents that belong to group *a* or *b*), positive values imply a process of sorting if neighbourhood makeup is defined in relation to the first group in the equation. Thus, if neighbourhood attribute *values* denote the percentage of residents belonging to group *a* in the neighbourhood, then ${\mathrm{DM}}_{\left(a,b\right)}^{i}>0$ implies a process of sorting; if they denote the percentage of residents belonging to group *b* in the neighbourhood, then $DM_{\left(a,b\right)}>0$ implies the opposite process of integration. For the sake of consistency, groups are ordered such that positive differential-mobility values imply a process of sorting. The larger the absolute value, the more intense the sorting. When mover sub-groups are ordinal with respect to social status, differential-mobility levels indicate to what extent higher-status groups upgrade their spatial position relative to lower-status groups. When increasing neighbourhood-attribute values reflect higher neighbourhood status (as in the variable ‘percentage of high-income residents’), the mobility level of a lower-status group *b* would be subtracted from that of a higher-status group *a* (*a* > *b*).

Trends in sorting can be assessed by computing measures for consecutive periods with the following interpretation:

I. $DM_{\left(t\right)}=DM_{\left(t+1\right)}$ a stagnant sorting process
II. $DM_{\left(t\right)}<DM_{\left(t+1\right)}$ an intensifying sorting process
III. $DM_{\left(t\right)}>DM_{\left(t+1\right)}$ a weakening sorting process

### Sorting across different geographical levels

Residential sorting occurs across different spatial levels. Imagine, for instance, an urban system with neighbourhoods that are nested within cities. Dominance of sorting at the city level is associated with larger variation in characteristics among cities, compared to the variation among neighbourhoods within these cities. In other words, the stronger the spatial autocorrelation when considering the neighbourhood level, the more dominant the sorting at the inter-city level will be. The extent to which mobility patterns translate into sorting among each level may change over time.

We posit that differential mobility calculated at the neighbourhood level $\left[DM_{\left(n\right)}\right]$, irrespective of city boundaries, is composed of two elements; the sorting among cities (differential-mobility measure computed using city-level characteristic values) $\left[{\mathrm{DM}}_{\left(c\right)}\right]$, and a remainder component which can be attributed to sorting among neighbourhoods within each city (Δ). This relationship resembles a separation that was suggested by Wong (2003) with regard to two segregation measures. While this article refers to cities and their constituent neighbourhoods, the separation may apply to other hierarchically nested geographical levels:

$$DM_{\left(n\right)}=DM_{\left(c\right)}+\Delta$$

An important thing to consider is the link between the spatial typology of relocations and the spatial level of sorting they generate. Sorting among cities occurs as a result of moves between them. If all relocations only occurred among neighbourhoods within each city, city-level disparities would not change as a result of mobility patterns. Sorting among neighbourhoods, on the other hand, can be a result of moves among neighbourhoods within cities and also of moves across cities. Moves from one city to another involve a change in both city- and neighbourhood-level attributes. The former may be represented as attributes averaged over all city neighbourhoods.

With no variation among neighbourhoods within each city, the mobility experienced at both the city- and neighbourhood-level would be identical; in the case of disparities among cities’ neighbourhoods, the level of mobility with respect to each level would vary. For example, if a person moved from the poorest neighbourhood in a poor city to the richest neighbourhood in a richer city, the change at the neighbourhood level perceived by this person would be higher than that of the city-level. The extent of sorting among neighbourhoods within cities, over and above the sorting generated among cities, is attributed to the extent of divergence of origin and destination neighbourhood characteristics from their respective city-averages. The separation of sorting measures to reflect contributions of relocations to the sorting at two spatial levels is based on that principle.

City attribute values in the destination ($d_{\left(c\right)}$) and origin ($o_{\left(c\right)}$) may be expressed as the averages of all constituent neighbourhoods’ attribute values:

$$\begin{array}{c} d_{\left(c\right)}={\bar{d}}_{\left(n\right)}^{c}\\ o_{\left(c\right)}={\bar{o}}_{\left(n\right)}^{c} \end{array}$$

Neighbourhood attribute values of the destination and origin ($d_{\left(n\right)}$ and $o_{\left(n\right)}$) may be expressed as the sum of the respective city-average values and the deviations of neighbourhoods’ values from the respective city-averages (denoted *Δd* and *Δo* for the destination and origin respectively):

$$\begin{array}{c} d_{\left(n\right)}=d_{\left(c\right)}+\Delta d\\ o_{\left(n\right)}=o_{\left(c\right)}+\Delta o \end{array}$$

The mobility measure can be used to reflect mobility at the city and neighbourhood levels (to simplify, measures here refer to individuals):

$$\begin{array}{c} M_{\left(c\right)}=d_{\left(c\right)}-o_{\left(c\right)}\\ M_{\left(n\right)}=d_{\left(n\right)}-o_{\left(n\right)} \end{array}$$

Where *M* is the mobility measure, *d* and *o* refer to attribute values at the destination and origin respectively and subscripts *c* and *n* refer to city and neighbourhood levels respectively. The mobility measure computed at the neighbourhood level, irrespective of its association with cities, may be expressed as the city-level mobility measure and an added component that emanates from the divergence of destination and origin characteristics from their respective city averages:

$$\begin{array}{c} M_{\left(n\right)}=\left(d_{\left(c\right)}+\Delta d\right)-\left(o_{\left(c\right)}+\Delta o\right)\\ =M_{\left(c\right)}+\Delta d-\Delta o \end{array}$$

The differential-mobility measure of two groups, ‘*a*’ and ‘*b*’, at the neighbourhood level can be written as:

$$\begin{array}{c} {\mathrm{DM}}_{\left(n\right)}^{a,b}=M_{\left(n\right)}^{a}-M_{\left(n\right)}^{b}=M_{\left(c\right)}^{a}-M_{\left(c\right)}^{b}\\ \;+\left(\Delta d^{a}-\Delta o^{a}\right)-\left(\Delta d^{b}-\Delta o^{b}\right)\\ \;(1)\;{\mathrm{DM}}_{\left(n\right)}^{a,b}={\mathrm{DM}}_{\left(c\right)}^{a,b}+\left(\Delta d^{a}-\Delta o^{a}\right)\\ \;-\left(\Delta d^{b}-\Delta o^{b}\right) \end{array}$$

Thus, it is shown that differential mobility calculated at the neighbourhood level is the sum of differential mobility calculated at the city level $\left({\mathrm{DM}}_{\left(c\right)}^{a,b}\right)$ and additional components relating to differences in the deviations of destination and origin neighbourhood attribute values from those of the respective city values, for groups *a* and *b*. These additional components can be ascribed to the sorting occurring among each city’s neighbourhoods.

**Figure 1.**
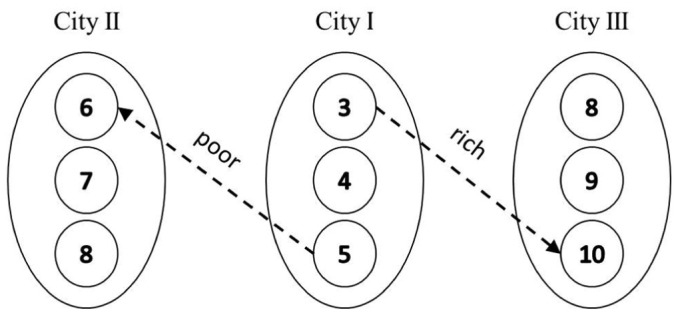
Relocations of a rich mover and a poor mover between neighbourhoods.

To illustrate, consider a hypothetical case of two movers, one rich and one poor (Figure 1), who both leave city I; the poor person moves to city II and the rich to city III. City and neighbourhood attribute values reflect socioeconomic status such that gaining higher values through relocation is interpreted as upward socio-spatial mobility. The rich and poor movers are superscripted ‘*a*’ and ‘*b*’ respectively.

Attribute values of cities I, II and III are averaged over each city’s neighbourhoods such that they are 4, 7 and 9 respectively. At the city level, socio-spatial mobility measures of the rich and poor movers are:

$$\begin{array}{c} M_{\left(c\right)}^{a}=d_{\left(c\right)}^{a}-o_{\left(c\right)}^{a}=9-4=5\\ M_{\left(c\right)}^{b}=d_{\left(c\right)}^{b}-o_{\left(c\right)}^{b}=7-4=3\\ {\mathrm{DM}}_{\left(c\right)}^{a,b}=M_{\left(c\right)}^{a}-M_{\left(c\right)}^{b}=2 \end{array}$$

The differential mobility of ‘*a*’ and ‘*b*’ at the city level therefore equals 2. Mobility measures can be computed at the neighbourhood level. Note that the poor mover moves from the richest neighbourhood in city I to the poorest neighbourhood in city II, while the rich mover moves from the poorest neighbourhood in city I to the richest in city III; neighbourhood-level disparities in socio-spatial mobility between the rich and poor movers are larger than city-level disparities, resulting in a differential measure of 6:

$$\begin{array}{c} M_{\left(n\right)}^{a}=d_{\left(n\right)}^{a}-o_{\left(n\right)}^{a}=10-3=7\\ M_{\left(n\right)}^{b}=d_{\left(n\right)}^{b}-o_{\left(n\right)}^{b}=6-5=1\\ {\mathrm{DM}}_{\left(n\right)}^{a,b}=M_{\left(n\right)}^{a}-M_{\left(n\right)}^{b}=6 \end{array}$$

Subtracting differential mobility at the city level from that of the neighbourhood level, we get the Δ component, which is attributed to the effect of relocations on sorting among neighbourhoods within each city:

$$\Delta ={\mathrm{DM}}_{\left(n\right)}^{a,b}-{\mathrm{DM}}_{\left(c\right)}^{a,b}=6-2=4$$

The contribution of relocations to sorting within cities is 4 percentage points, which amounts to 66% of the total measure of sorting.

In the above example, the deviation in city- and neighbourhood-level contributions to sorting emanates from the way mobility patterns are structured, with the poor and rich switching their neighbourhoods’ city-relative positions. Consider example (2); if the poor and rich movers moved from the poorest and richest neighbourhoods of city I respectively, the Δ component would be 0, reflecting an identical deviation of destination and origin neighbourhoods from city averages. Their moves would not change the within-city component of sorting because they preserved city-relative positions. Consider again example (2), but with larger deviations among destination-city neighbourhoods from city averages, such that city II and city III neighbourhood values were (5,7,9) and (7,9,11). In this case, the Δ component would equal 2 due to a larger variation among cities’ neighbourhoods in relation to the variation among cities (weaker spatial autocorrelation). With perfect spatial autocorrelation (i.e. cities vary but neighbourhoods within cities do not), the Δ component would again equal 0 regardless of the mobility structure. The spatial level of sorting is influenced, therefore, both by the mobility structure and by the existing socio-spatial structure.

## An empirical demonstration

The empirical demonstration explores trends in income sorting in the metropolitan area of Tel-Aviv, Israel, between 1997 and 2008. The database includes yearly individual moving records with geographical identification of origin and destination census tracts and their matched yearly socioeconomic attributes. The data is based on a 50% sample (stratified according to places of origin and random with respect to individual attributes) of individual intra-metropolitan moving records (N = 699,793), including movers’ income decile,^4^ processed by the Israeli Central Bureau of Statistics (CBS) and based on governmental address-change listings. Appended census tract attributes (908 in total) are based on census data from 1995 and 2008 (CBS), with linearly interpolated data for inter-censual years. We represent neighbourhood socioeconomic status by the percentage of high-income residents, defined as those pertaining to the three highest income deciles in each year respectively.

### Trends in socio-spatial mobility and sorting

**Figure 2.**
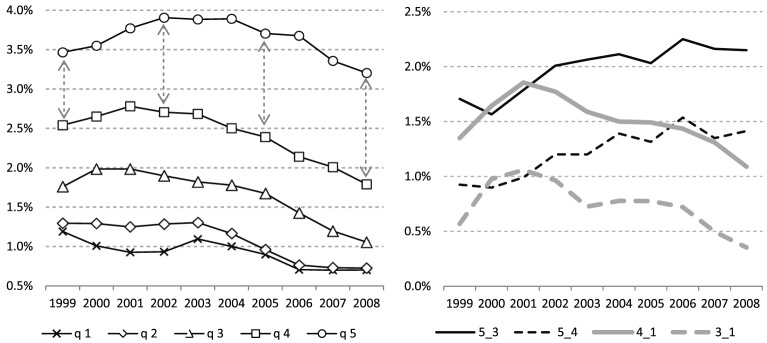
Left panel: Per-capita mobility (three-year moving average) according to movers’ income quintile. Operationalised as the mean origin–destination difference in variable values (percentage points). Right panel: Differential mobility (three-year moving average) – highest (5th) and lowest (1st) quintiles relative to middle quintiles (3rd and 4th).

Socio-spatial mobility measures for each income quintile (1 = lowest incomes) indicate that movers of quintiles 1–4 experienced relatively stable levels and then declines in upward socio-spatial mobility (Figure 2, left panel). The 5th quintile experienced, on average, increasing upward mobility until 2002 and then moderate decreases. The increasing divergence between quintiles 4 and 5, for example, is signified by arrows with increasing lengths. The changing divergence is further examined among other quintile pairs using the ‘differential mobility’ measure of sorting (right panel).

Differential-mobility measures were computed for the highest and lowest quintiles (5th and 1st quintiles, respectively) relative to the middle and middle-high quintiles (3rd and 4th) (Figure 2, right panel). The lowest quintile has experienced increasing disparities relative to middle quintiles during a short initial period, after which they steadily declined; disparities between the highest and middle quintiles have steadily grown throughout the research period.

### Trends in sorting at neighbourhood and city levels

**Figure 3.**
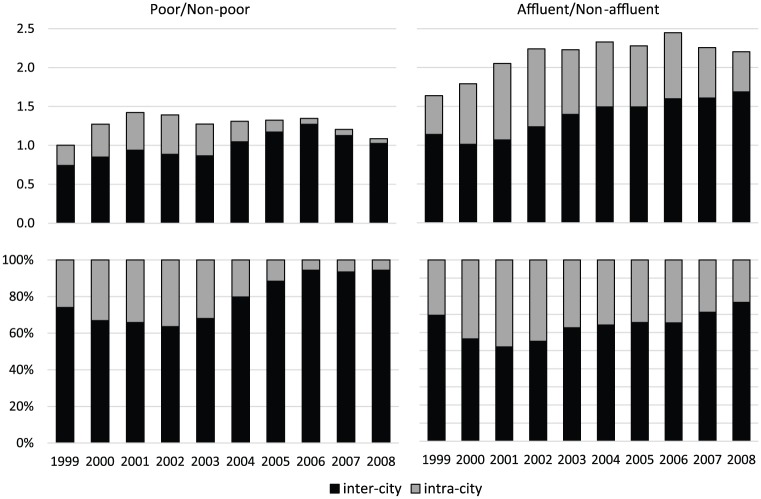
Absolute (upper panels) and percentage (lower panels) contributions of inter- and intra-city levels to inter-group differential-mobility measures. Lowest (left panels) and highest (right panels) quintiles relative to all other quintiles.

This analysis was performed using a subsample of relocation records (87% of the total mobility sample), in which origins and destinations are neighbourhoods that are hierarchically nested within cities. Differential measures were computed for the lowest and highest quintiles, each relative to all other quintiles (aggregated together). Measures were broken down to reflect the relative contributions of sorting among cities and among neighbourhoods within these cities (Figure 3). The separation indicates that residential mobility patterns have increasingly contributed to income sorting among cities, at the expense of sorting within cities. With respect to the poorest movers, the sorting among cities accounts towards the end of the research period for about 95% of the total measure of sorting (lower-left panel). Sorting within cities was more pronounced with respect to sorting of the highest quintile; but likewise, its relative share of the total measure has diminished from a maximum of about 50% to a minimum of 23% in a few years (lower-right panel).

## Conclusions

The purpose of this article is to develop a methodological framework designed to explore how changing residential mobility patterns reflect temporal and scale variations in sorting. Current practices in the analysis of residential mobility cannot advance this understanding because they do not quantify the changing divergence in mobility patterns of different mover groups and do not deal with how this changing divergence is structured across different spatial levels.

This article introduces a measure of sorting that can indicate the changing extent and scale of sorting reflected in mobility patterns. It is based on a socio-spatial mobility approach which regards residential relocations between neighbourhoods as movement across social positions and focuses on quantifying such moves. We derive the sorting measure by quantifying disparities among mover sub-groups in their socio-spatial mobility. Sorting measures computed over consecutive periods reveal trends in the direction and extent of sorting. By computing it using hierarchically nested geographical units, for example cities and their constituent neighbourhoods, the measure of sorting can be broken down to components that reflect the relative contributions of mobility to sorting at these spatial levels. The proposed measure of sorting indicates whether the tendency to sort at each time point has increased relative to the previous period. It is important to notice that this measure does not indicate resulting spatial outcomes of sorting, but rather summarises a marginal effect of moving behaviours.

The empirical demonstration in this article shows how changing patterns of mobility in the Tel-Aviv metropolitan area in Israel translate into an increasing tendency of the affluent, coupled with a decreasing tendency of the poor, to segregate from middle-income groups, throughout the study period (1997–2008). Sorting processes have also changed with respect to the spatial level; residential mobility patterns increasingly contributed to sorting at the inter-city rather than the within-city level in the metropolitan area. The example confirms that the magnitude and spatial scale of residential sorting change, even over the short term.

## Notes

1. Poor neighbourhoods were classified by Crowder and South (2005) as those with poverty rates above 20% and by Gramlich et al. (1992) as those with poverty rates exceeding 30%. ‘Black’ neighbourhoods in the US were classified by Massey et al. (1994) using a 60% threshold while in Quillian (2002) and South and Crowder (1998) thresholds were 70% and 89% respectively.

2. The approach has been used in the socioeconomic context but can also be used in an ethnic context.

3. The use of absolute measures should better be restricted to data representing relocation event records, rather than data based on individuals’ locations retrieved from census years which are collected years apart. Otherwise, structural changes can be captured as part of the change attached to the relocation itself.

4. Based on national income distributions for each year.
